# Plasticity of *Streptomyces coelicolor* Membrane Composition Under Different Growth Conditions and During Development

**DOI:** 10.3389/fmicb.2015.01465

**Published:** 2015-12-22

**Authors:** Mario Sandoval-Calderón, Don D. Nguyen, Clifford A. Kapono, Paul Herron, Pieter C. Dorrestein, Christian Sohlenkamp

**Affiliations:** ^1^Centro de Ciencias Genómicas, Universidad Nacional Autónoma de MéxicoCuernavaca, Mexico; ^2^Department of Chemistry and Biochemistry, University of California, San Diego, La JollaCA, USA; ^3^Strathclyde Institute of Pharmacy and Biomedical Sciences, University of StrathclydeGlasgow, UK; ^4^Skaggs School of Pharmacy and Pharmaceutical Sciences, University of California San Diego, La JollaCA, USA

**Keywords:** *Streptomyces*, bacterial development, phospholipids, ornithine lipids, phosphorus limitation, membrane adaptation

## Abstract

*Streptomyces coelicolor* is a model actinomycete that is well known for the diversity of its secondary metabolism and its complex life cycle. As a soil inhabitant, it is exposed to heterogeneous and frequently changing environmental circumstances. In the present work, we studied the effect of diverse growth conditions and phosphate depletion on its lipid profile and the relationship between membrane lipid composition and development in *S. coelicolor*. The lipid profile from cultures grown on solid media, which is closer to the natural habitat of this microorganism, does not resemble the previously reported lipid composition from liquid grown cultures of *S. coelicolor*. Wide variations were also observed across different media, growth phases, and developmental stages indicating active membrane remodeling. Ornithine lipids (OL) are phosphorus-free polar lipids that were accumulated mainly during sporulation stages, but were also major components of the membrane under phosphorus limitation. In contrast, phosphatidylethanolamine, which had been reported as one of the major polar lipids in the genus *Streptomyces*, is almost absent under these conditions. We identified one of the genes responsible for the synthesis of OL (*SCO0921*) and found that its inactivation causes the absence of OL, precocious morphological development and actinorhodin production. Our observations indicate a remarkable plasticity of the membrane composition in this bacterial species, reveal a higher metabolic complexity than expected, and suggest a relationship between cytoplasmic membrane components and the differentiation programs in *S. coelicolor*.

## Introduction

*Streptomyces coelicolor* is a soil dwelling actinomycete that has been for decades a model for the study of secondary metabolism and differentiation in Gram-positive bacteria. It displays a complex life cycle that involves both morphological and physiological differentiation. The cycle begins with germination of unicellular spores, followed by hyphal outgrowth leading to formation of a vegetative mycelium. In the next phase during development, aerial hyphae escape the surface tension of the aqueous environment, and later differentiate into chains of spores whose main role is dispersion and that have limited resistance to stresses (Flardh and Buttner, 2009). Morphological differentiation is a coordinated process that involves an interplay between environmental stimuli such as nutritional cues, physiological conditions and extracellular signaling (McCormick and Flärdh, 2012). Nutritional downshift seems to be a trigger for morphological differentiation, responding to a drop in GTP pool (Ochi, 1986; Okamoto and Ochi, 1998) and to ppGpp synthesis (Hesketh et al., 2007; Tschowri et al., 2014). Diverse carbon sources (*N*-acetylglucosamine, glucose, mannitol, or galactose) also elicit differences in development (Champness, 1988; Pope et al., 1996; Rigali et al., 2006). Cyclic dinucleotide c-di-GMP has been shown to act as a second messenger to control differentiation through the activation of DNA binding of transcriptional regulator BldD (Tschowri et al., 2014). Extracellular signaling is also relevant in different *Streptomyces* species; A-factor is a well-studied γ-butyrolactone that regulates both morphological differentiation and antibiotic biosynthesis in *Streptomyces griseus* (Horinouchi et al., 2001). Related γ-butyrolactones in *S. coelicolor* can also influence development (D’Alia et al., 2011). In addition, the products of extracellular proteolytic activity may also be part of the complex signaling network underlying the coordination of morphological development (Chater et al., 2010).

The bacterial cytoplasmic membrane has a major role in cellular functions, both as a physical barrier that delimits the cellular interior and as a structural component where many of the core metabolic activities are organized, but membranes are also active players in metabolism and interaction with the environment. The composition of biological membranes must adapt to diverse conditions in order to maintain their functions and cope with the challenging extracellular environment (Zhang and Rock, 2008). Bacteria are able to remodel the fatty acid content of their phospholipids to deal with changes in temperature or the presence of membrane disrupting compounds (Sinensky, 1974; Ingram, 1977); several species synthesize new membrane molecules as a response to antimicrobial peptides (Roy, 2009); some replace their phospholipids with phosphorus-free polar lipids as a result of inorganic phosphate starvation (Geiger et al., 1999); others modify their membrane components to cope with acidic (Sohlenkamp et al., 2007) and osmotic stress (López et al., 1998; Romantsov et al., 2009; Lopalco et al., 2013) or to interact with their hosts (Tannaes et al., 2005).

**FIGURE 1:**
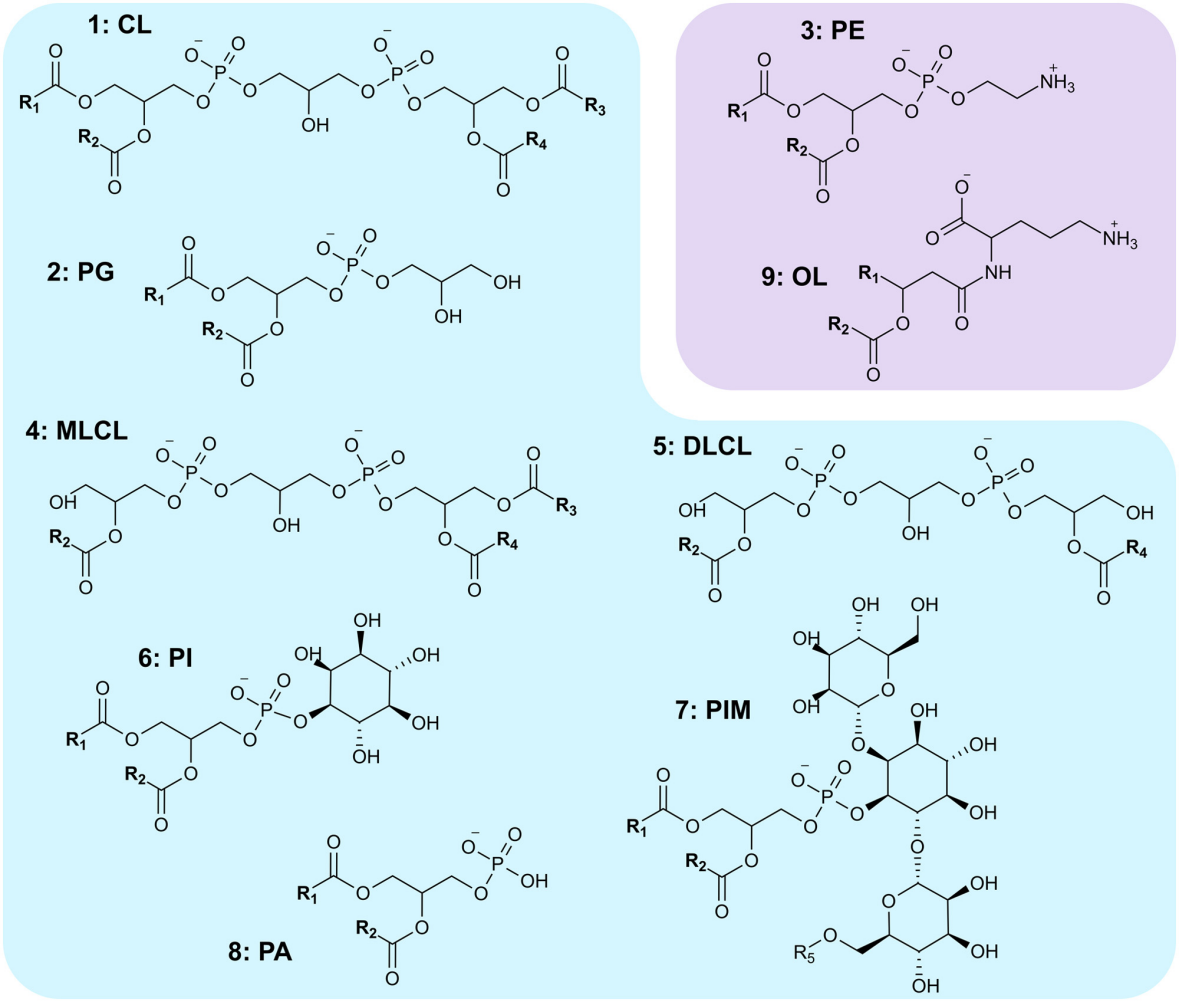
**Polar lipids synthesized by *Streptomyces coelicolor*.** **(1)** CL, cardiolipin; **(2)** PG, phosphatidylglycerol; **(3)** PE, phosphatidylethanolamine; **(4)** MLCL, monolyso-cardiolipin; **(5)** DLCL, dilyso-cardiolipin; **(6)** PI, phosphatidylinositol; **(7)** PIM, phosphatidylinositol mannosides; **(8)** PA, phosphatidic acid; **(9)** OL, ornithine lipid. R_1-4_ represent acyl chains, R_5_ represents either an H atom or an acyl chain. Lipids shaded in blue are anionic; those shaded in purple are zwitterionic.

The biosynthesis and lipid composition of the cytoplasmic membrane of *S. coelicolor* remain largely unexplored. We recently found that *S. coelicolor*, along with most actinomycetes, synthesizes CL (**1**) through a pathway previously thought to be exclusive for eukaryotes (Sandoval-Calderón et al., 2009). This biosynthetic difference probably drives the singular phospholipid profile from streptomycetes, which have a very high CL (**1**) to PG (**2**) ratio (Lechevalier et al., 1977; Zuñeda et al., 1984; Hoischen et al., 1997; Sandoval-Calderón et al., 2009) compared to most bacteria. We also described the presence of PE (**3**), MLCL (**4**), DLCL (**5**), PI (**6**), and PIMs (**7**) in *S. coelicolor* membranes (**Figure 1**) (Sandoval-Calderón et al., 2009). In a later study by Jyothikumar et al. (2012), evidence for accumulation of PG and PA (**8**) is also presented.

It was recently reported that cardiolipin synthase (ClsA) is an essential enzyme in *S. coelicolor*, and that appropriate expression of ClsA was needed for aerial hyphae erection (Jyothikumar et al., 2012). It has been also found that inositol synthesis (and putatively, PI) is necessary for correct morphological development in this same organism (Zhang et al., 2012). However, the role that different membrane components could play during *Streptomyces* morphological differentiation is still not understood.

We hypothesize that *S. coelicolor* is able to remodel its membrane in order to adapt to the environment and as a result to its morphological development program. In the present work, we studied the lipid composition of *S. coelicolor* under a diverse set of growth conditions. *S. coelicolor* undergoes radical alterations of its membrane components as a result of development, inorganic phosphate availability, and growth in liquid vs. solid media. Furthermore, we identify genes responsible for biosynthesis of OL (**9**), which becomes a major component of the membrane in some of the growth conditions explored. Our results show that the complexity and dynamics of the lipid composition of *Streptomyces* had previously been greatly underestimated.

## Materials and Methods

### Media, Strains and Growth Conditions

*Streptomyces coelicolor* M145 was used as wild-type strain. Inactivation of *SCO0921* was performed following the last steps of the PCR-directed mutagenesis protocol described by Gust et al. (2003) using the mutagenized cosmid SCM10.1.C05 that carries an insertion of transposon Tn*5062* (Bishop et al., 2004) toward the end of the sequence from *SCO0921*.

To perform a complementation assay, a *SCO0921* mutant strain was constructed that expressed in *trans* the putative operon comprised by *SCO0921-SCO0920*. Amplification of *SCO0921-SCO0920* was done with primers 5′-ATATGGATCCGCGTTCAGTGAACGTTCGCC-3′ and 5′-ATTAGAATTCATCCAGCGGTATGGAAACGGC-3′ using AccuPrime^TM^ *Pfx* polymerase. The resulting 1.8 kb sequence includes 200 base pairs upstream of *SCO0921*. This PCR product was cloned into pBluescript II SK+ (Stratagene) using *Eco*RI and *Bam*HI restriction sites included in the primers. This construction was cointegrated with pIJ6902 expression vector after digestion of both vectors with *Eco*RI to obtain plasmid pIJ-*olsBA*. A cointegrate between pBluescript II SK+ and pIJ6902 was constructed the same way to use as a negative control, the resulting vector was named pIJ-BS. Both plasmids were mobilized into *S. coelicolor* M145 and the *SCO0921*-deficient mutant as described by Gust et al. (2003) using *Escherichia coli* ET12567.pUZ8002 as the donor strain.

To collect samples from *S. coelicolor* cultures undergoing development, most experiments in solid media were performed with mycelium scraped from cellophane disks overlaid on the surface of agar plates as described elsewhere (Kieser et al., 2000). Autoclaved wet cellulose acetate films were placed on top of the agar and dried in a laminar flow cabinet. Plates were then inoculated with 10^7^ spores from a concentrated spore stock. The media used were: YEME liquid (34% sucrose) and solid medium (30% sucrose), Supplemented Minimal Medium Solid (SMMS), SFM, R2YE (Kieser et al., 2000) and ISP2 (Shirling and Gottlieb, 1966). R2YE with 0.05, 0.5, or 5 mM of KH_2_PO_4_ as inorganic phosphate (P_i_) source was used when indicated. Cultures were incubated at 30°C between 1 and 9 days, as indicated in the results section. ISP2 and YEME are normally used for routine growth of *S. coelicolor*, while SFM is used to promote rapid sporulation. SMMS and R2YE are widely used for studies on antibiotic production. Minimal media were excluded from the analysis due to the low yield of lipids extracted from the samples as a consequence of the reduction of biomass.

### Lipid Extraction

Mycelium samples were recovered from agar plates by scraping the surface of the cellophane disks with a spatula. Total biomass collected was weighed and suspended in enough water to reach 1 or 2 ml. Samples were stored at -20°C. After thawing, lysozyme was added to the samples to a final concentration of 2 mg/ml and they were incubated for 1 h at 37°C. Lipid extraction was then performed according to Bligh and Dyer (1959). After drying the chloroform phase, each sample was dissolved in a volume of chloroform/methanol (1:1) proportional to the amount of wet biomass of the mycelium from which it was derived (10 μl of solvent per 30 mg of mycelium).

### Thin Layer Chromatography Analysis

Two different TLC systems were used for the separation of polar lipids. System 1 was performed on high-performance TLC aluminum sheets (Silica Gel 60, Merck), with a chloroform/methanol/water (16:4:1, v/v) mixture used for the first dimension, and chloroform/methanol/acetic acid (15:3:2, v/v) for the second dimension (Tahara et al., 1994). However, this system for TLC separation was inadequate for complete separation of two major phospholipids of *S. coelicolor*, DLCL and PI (Sandoval-Calderón et al., 2009). Therefore, an improved method was adapted from a system for one-dimensional TLC (Vaden et al., 2005) separation. Before sample application, HP-TLC plates were soaked in a solution of 1.8% boric acid in ethanol, dried for 5 min, and then baked for 15 min at 100°C. The solvent mixtures used to develop the chromatography were chloroform/methanol/water (16:4:1, v/v) for the first dimension and chloroform/ethanol/water/triethylamine (30:35:7:35 v/v) for the second dimension. After developing in two dimensions, TLC plates were dried overnight and then developed again in the second dimension. This improved system is referred to as system 2.

The amount of lipids extracted from approximately 35 mg of mycelium (∼12 μl of lipid preparation) was usually enough to have a proper visualization of the major lipid classes. However, for samples derived from cultures more than 4 days old, the volume of sample loaded was increased 1.5-fold.

### Lipid Visualization and Quantification

To detect hydrophobic molecules, TLC plates were sprayed with ANS reagent (8-Anilino-1-naphthalenesulfonic acid, Sigma) at 0.2% in methanol (Zbierzak et al., 2011) and lipids were visualized as fluorescent spots under UV light (366 nm). Photographs of TLC plates were processed with ImageJ (Schneider et al., 2012) as follows: contrast was increased; images were converted to black and white and inverted for easier visualization. In order to obtain a rough quantification of the lipids, ImageJ was used to measure the integrated density of each spot from the ANS-stained TLC plates. Spots corresponding to neutral lipids were not included in the quantification analysis.

In some experiments, 2D-TLC plates were stained with ninhydrin (Sigma), Phospray reagent (Supelco), or orcinol (Sigma) for detection of aminolipids, phospholipids, and glycolipids, respectively. Identity of the different lipids was assigned by comparison with R_f_ from lipid standards analyzed in the same TLC systems (**Supplementary Figure S1**), coupled with the use of different TLC stainings. Identification of some molecules was also complemented with MS analyses.

### Mass Spectrometry and MS/MS Networking

Selected lipid samples were diluted 1:100 in methanol with 1 mM ammonium acetate to aid in the ionization of both anionic and zwitterionic lipids, then directly infused into a 6.42 T Thermo LTQ-FT-ICR mass spectrometer using a Triversa nanomate-electrospray ionization source (Advion Biosystems). Electrospray ionization was performed with a spray voltage of 1.3-1.45 kV and a back pressure of 0.35-0.5 psi. MS/MS spectra were acquired using softwares Tune Plus version 1.0 and Xcalibur version 1.4 SR1. The instrument was first tuned to 816 *m/z*, using cytochrome C ([M + 15H]^15+^, Sigma Aldrich). Data was collected using a data-dependent MS/MS method in the positive ion mode. The method consisted of one 10 min segment, during which a profile FT-MS scan within a range of 50-1,600 *m/z* was iteratively cycled with MS/MS scans of the most intense ions in the ion trap (Yang et al., 2013).

An MS/MS network of the hydrophobic molecules from *S. coelicolor* was constructed using the data analysis workflow at the Global Natural Products Social Molecular Networking site (GNPS^1^) with the combined results of all the MS data obtained from the different samples of *S. coelicolor* lipid extracts. All files used in the construction of the network were deposited in the MassIVE public GNPS database (MassIVE ID: MSV000078623). The parameters used for the network construction were: a precursor mass tolerance of 2.0 Da, ion tolerance of 0.5 Da, minimum cluster size of 2, maximum connected component size of 100, Network TopK of 10, maximum shift mass of 100 Da, cosine threshold was set at 0.7, and a minimum of two matched peaks was required. The resulting network was then visualized with Cytoscape version 2.8.3^2^ using the FM3 layout. MS/MS data from extracts of culture media were also included in the network construction, and the corresponding nodes were then eliminated from the network to reduce background signals.

## Results

### The Lipid Profiles of *S. coelicolor* Grown in Liquid vs. Solid Media are Different

In their natural habitats streptomycetes normally grow either on solid surfaces, or at the water/air interphase in soil, and several species, including *S. coelicolor*, are not able to undergo all stages of development when grown in liquid media. Metabolic differentiation can also be altered by growth in submerged cultures. The previous characterizations of the *S. coelicolor* lipid profile had been performed in the latter conditions (Sandoval-Calderón et al., 2009; Jyothikumar et al., 2012), therefore we wondered if *S. coelicolor* grown on solid medium would present a different membrane lipid composition compared to bacteria grown in liquid medium.

**FIGURE 2:**
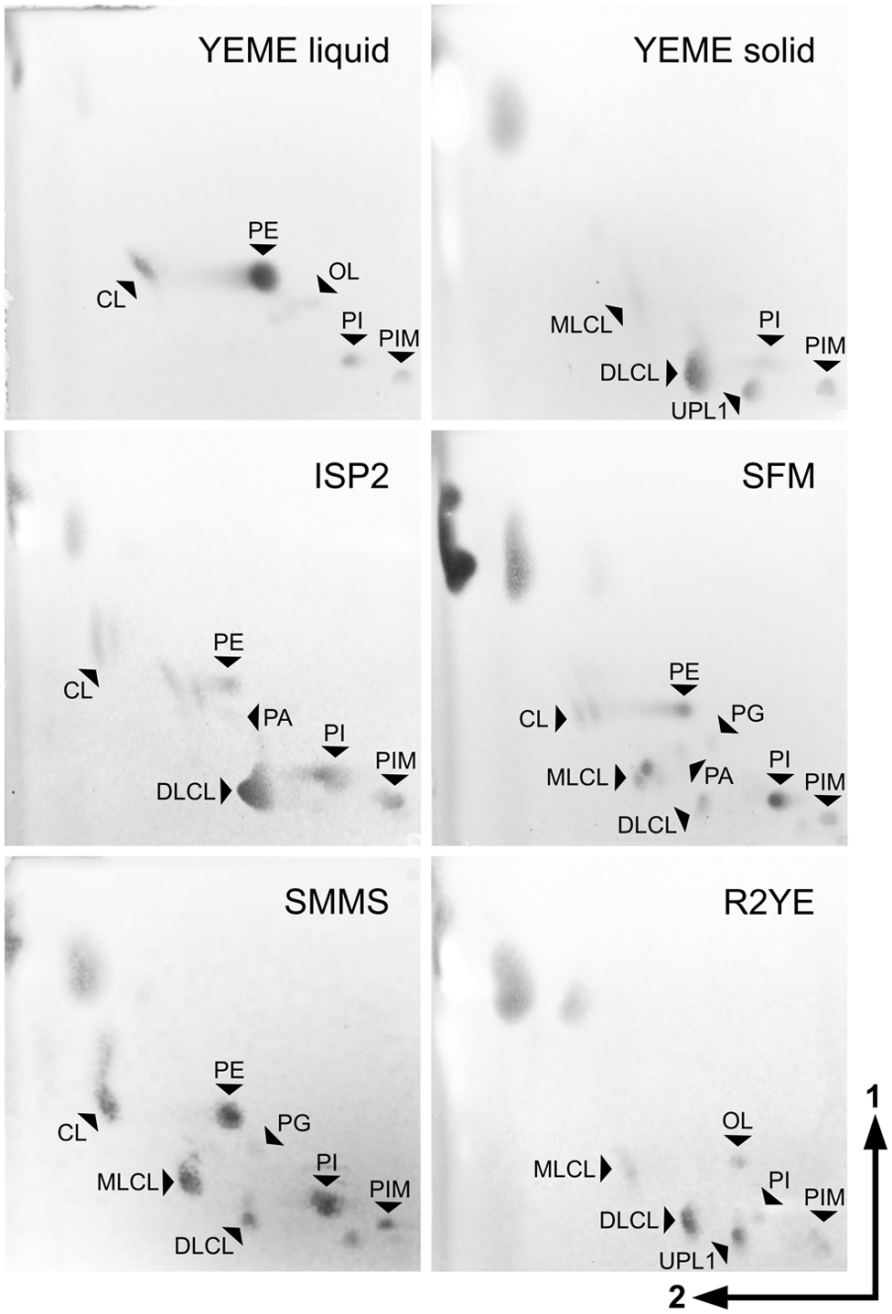
**Plasticity of *S. coelicolor* membrane lipid composition.** Lipid extracts of *S. coelicolor* M145 samples grown in different media were analyzed by TLC. Mycelium grown in YEME liquid medium was harvested at mid exponential phase. Vegetative mycelium from solid media was harvested just before the onset of aerial hyphae erection, between 2 and 4 days after inoculation (3 days for YEME solid medium, 4 days for ISP2, 2 days for SFM, 2 days for SMMS, and 40 h for R2YE). TLCs were developed with system 2, stained with ANS reagent to reveal all hydrophobic molecules and visualized under UV light. Identification of the spots was performed by comparison with lipid standards analyzed in the same TLC system (**Supplementary Figure S1**). PE, phosphatidylethanolamine; CL, cardiolipin; MLCL, monolyso-cardiolipin; DLCL, dilyso-cardiolipin; PI, phosphatidylinositol; PIM, phosphatidylinositol mannosides; PG, phosphatidylglycerol; PA, phosphatidic acid; OL, ornithine lipid; UPL1, unknown phospholipid.

In cultures harvested during exponential growth in YEME liquid medium, the most abundant lipid species were PE (**3**) and CL (**1**), with minor amounts of PI (**6**) and PIMs (**7**) (**Figure 2**, top left panel) as previously reported (Sandoval-Calderón et al., 2009). In contrast, *S. coelicolor* grown in solid YEME medium presented barely detectable amounts of PE (**3**) and CL (**1**), and accumulated instead MLCL (**4**) and high amounts of DLCL (**5**) – putative derivatives of CL (**1**) –, in addition to PIMs (**7**), PI (**6**) and an unidentified phospholipid (**Figure 2**, top right panel).

### The Lipid Profile from *S. coelicolor* Varies Dramatically in Different Culture Media

**FIGURE 3:**
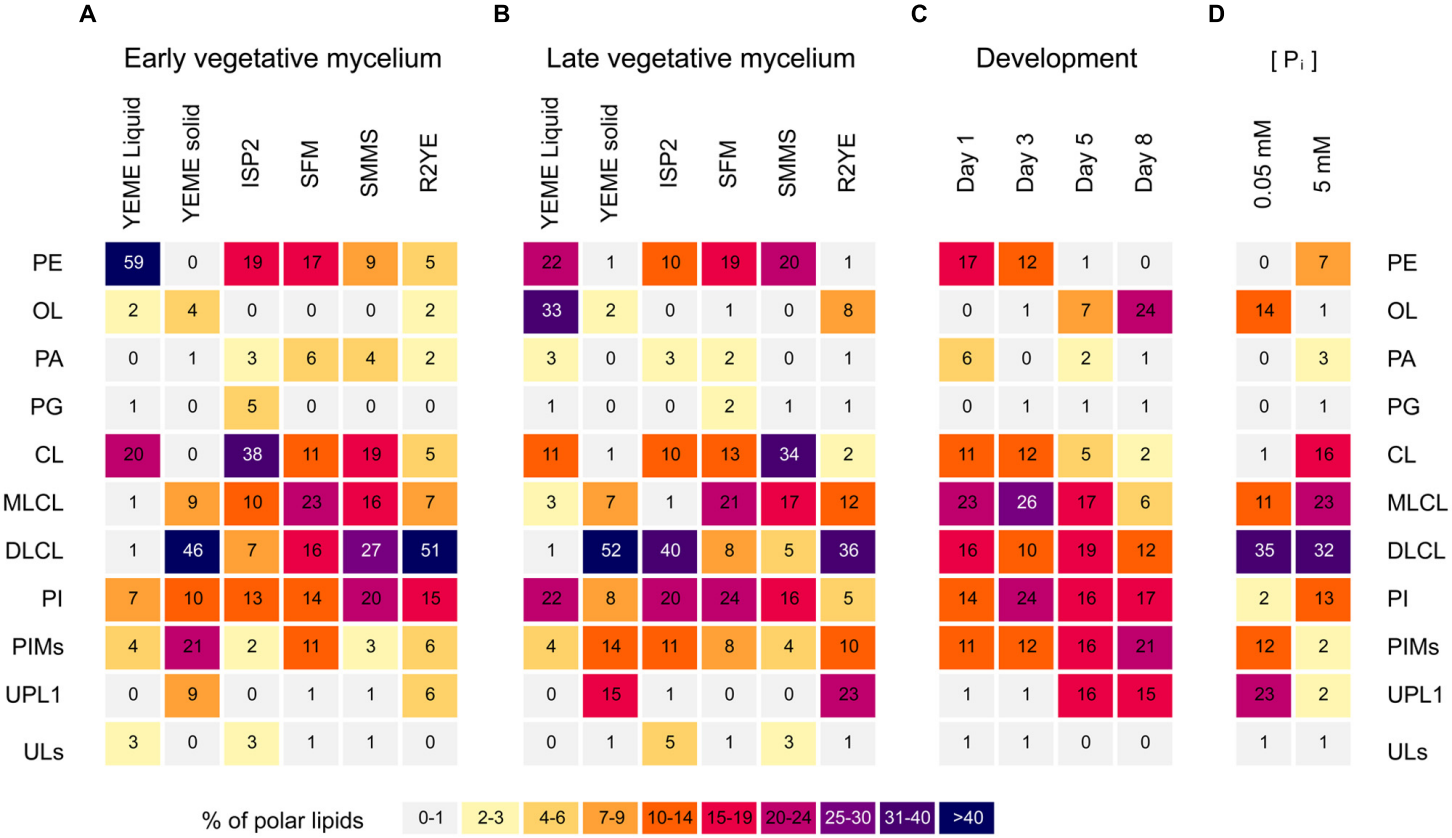
**Heatmap of the polar lipid composition from *S. coelicolor*.** Cultures were grown in different media and samples were taken throughout different developmental phases. **(A)** Samples of early vegetative mycelium were collected at early time points during growth, before beginning of prodiginine accumulation. Liquid cultures in YEME were harvested at mid-exponential phase. Cultures grown in solid media were harvested few days after inoculation: 2 days for YEME, 1 day for ISP2, SFM, SMMS, and R2YE. Differences in harvest times were due to the different growth rates in each medium. **(B)** Samples of late vegetative mycelium were harvested at stationary phase in YEME liquid medium, or just before the onset of aerial hyphae erection in cultures grown in solid media: 3 days for YEME, 4 days for ISP2, 48 h for SFM and SMMS, and 40 h for R2YE. **(C)** Lipid composition throughout development in SFM medium. Samples were taken 1, 3, 5, or 8 days after inoculation, comprising vegetative mycelium, mycelium producing aerial hyphae, mycelium beginning sporulation, and mycelium undergoing plentiful sporulation, respectively. **(D)** Cultures grown in R2YE solid medium for 60 h under phosphate limitation (0.05 mM P_i_) or repletion (5 mM P_i_). CL, cardiolipin; MLCL, monolyso-cardiolipin; DLCL, dilyso-cardiolipin; PE, phosphatidylethanolamine; OL, ornithine lipid; PA, phosphatidic acid; PG, phosphatidylglycerol; PI, phosphatidylinositol; PIMs, phosphatidylinositol mannosides; UPL1, unknown phospholipid; ULs, other unknown polar lipids.

*Streptomyces coelicolor* was grown on other complex solid media such as ISP2, SFM, SMMS and R2YE. Samples from vegetative mycelium were isolated; lipids were extracted and analyzed by TLC (**Figures 2** and **3A,B**). The lipid profiles from vegetative mycelium were similar between samples grown in SMMS and SFM, presenting more balanced amounts of CL (**1**), MLCL (**4**), DLCL (**5**), PE (**3**) and PI (**6**) than the other samples. The distribution of polar lipids in R2YE samples was akin to that of samples from YEME solid medium, containing DLCL (**5**) as the dominant phospholipid and little accumulation of PE (**3**) and CL (**1**) in contrast to the rest of cultures (**Figures 2** and **3A,B**). A common characteristic between cultures in YEME and R2YE solid media was abundant and early pigment production (data not shown).

Two different time points from vegetative mycelium were compared, samples collected before accumulation of pigments (early vegetative mycelium), and samples harvested before the onset of aerial hyphae erection (late vegetative mycelium). Early vegetative mycelium grown in ISP2 shared several similarities with SFM, such as the amount of PE (**3**) and PI (**6**) accumulated (**Figure 3A**). However, samples from late vegetative mycelium in ISP2 were more similar to solid YEME samples, presenting a remarkable accumulation of DLCL (**5**) (**Figure 3B**) and prodiginines (not shown). PA (**8**) and PG (**2**) were found as minor components of the lipid profile in most media, but in slightly higher amounts in samples from early vegetative mycelium (**Figures 3A,B**), particularly in SFM and SMMS media. OL (**9**) was predicted to be produced by *S. coelicolor* (Sohlenkamp and Geiger, 2015), but experimental evidence for its synthesis by this species was lacking. TLC spots corresponding to a phosphorus-free aminolipid that were assigned as OL were found as minor components of the lipid profiles from early cultures in YEME and R2YE (**Figure 3A**). This putative OL was a major lipid in mycelium samples beginning stationary phase from YEME liquid cultures (**Figure 3B**). Additionally, in late vegetative mycelium, there was accumulation of an unknown phospholipid with an R_f_ close to that of PI in cultures from R2YE and YEME solid media (**Figures 2** and **3B**).

### Mass Spectral Molecular Networking from Lipid Samples of *S. coelicolor* Highlights Metabolic Differences in Different Media and Developmental Phases

**FIGURE 4:**
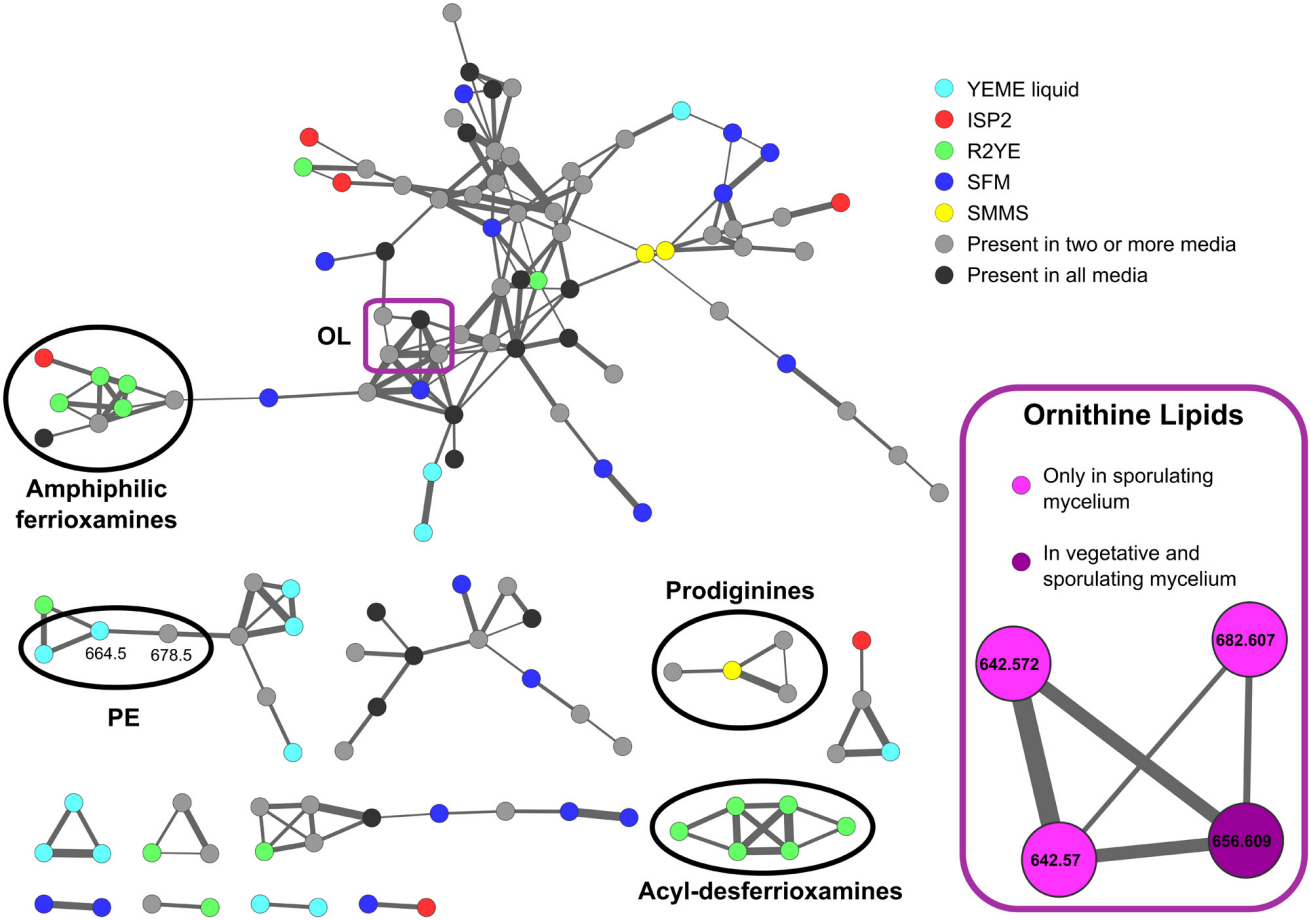
**Molecular network of *S. coelicolor* lipid extracts.** Cultures were grown in liquid YEME medium, and solid ISP2, R2YE, SFM, and SMMS media. The sample from liquid YEME was harvested at mid-exponential phase. Mycelium samples from solid media were taken at the vegetative phase in SMMS, and R2YE, and across the three main stages of development in SFM and ISP2. Samples of aerial and sporulating mycelium from SMMS and R2YE were not included in the network because the extremely high amount of prodiginines produced in these conditions obscured the signals from polar lipids. Nodes represent different precursor ions in the samples, and edges between the nodes represent the similarity between fragmentation spectra of the corresponding precursor ions. The thickness of the edges is proportional to the cosine score between the MS/MS spectra. Node colors denote different culture media or the time during development where a particular molecule was found (in the sub-network highlighted in the inset). Several clusters are highlighted where annotation of the molecular ions was possible. Annotation was performed based on the cosine scores with matches from compounds in the GNPS library and comparison of their MS/MS profiles, or by comparison with MS/MS fragmentation reported in the literature. PE, phosphatidylethanolamine; OL, ornithine lipids.

To complement the results obtained by TLC, we analyzed some lipid samples using MS. The MS/MS fragmentation data was organized with the Mass Spectral Molecular Networking methodology (Watrous et al., 2012). The molecular network highlighted several differences in the metabolic profile between the samples being considered (**Figure 4**), with many molecular ions detected only in specific media, and relatively few shared across all the samples. Some of the precursor ions corresponding to PE (**3**) (664.5 *m/z*) were only present in samples from cultures harvested from YEME liquid medium, consistent with the high content of PE in these samples. Other PE ions, like 678.5 *m/z* were found across several media. A subcluster of the largest part of the network (**Figure 4**, inset) was highlighted because its *m/z* values (and corresponding MS/MS spectra) match those expected for OLs (**9**) (642.6, 656.6, 682.6 *m/z*) (**Figure 1**). Most of OL ions were detected at later stages during development in solid media.

Most of the other metabolites, ranging from 200 *m/z* to 1500 *m/z*, could not be completely identified. However, some clusters within the network match the fragmentation profiles of molecules contained in the GNPS libraries, namely, those corresponding to undecylprodigiosin (CCMSLIB00000072310), streptorubin (CCMSLIB00000072253), and several acyl-desferrioxamines (Traxler et al., 2013) (C10: CCMSLIB00000072043; C11: CCMSLIB00000072046; C12: CCMSLIB00000072049; C13: CCMSLIB00000072052; C14: CCMSLIB00000072054). Interestingly, the cluster corresponding to the acyl-desferrioxamines was only found in samples from R2YE solid medium. The prodiginines, on the other hand, were found in ISP2, R2YE and SMMS solid media, although streptorubin B (392.27 *m/z*) was only detected in SMMS (**Supplementary Figure S2**).

### The Membrane Lipid Composition of *S. coelicolor* is Dynamic During Development

**FIGURE 5:**
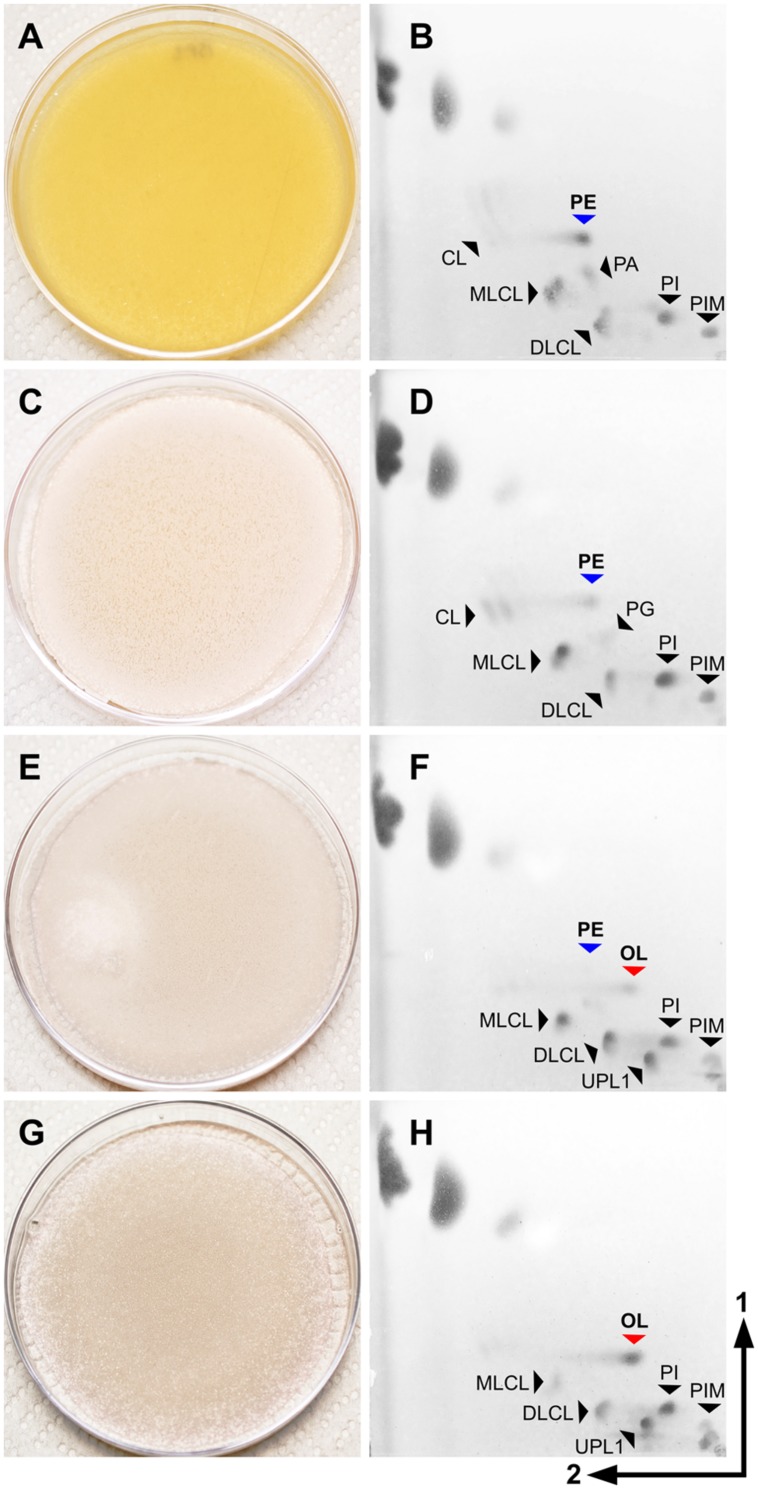
**Membrane lipid composition in *S. coelicolor* is altered during development.** All cultures were grown in solid SFM medium **(A,C,E**, and **G)**. Lipid profiles were analyzed by 2D-TLC **(B,D,F**, and **H)**. TLC plates were developed with system 2, stained with ANS reagent to reveal all hydrophobic molecules and visualized under UV light. **(A**,**B)** 1 day after inoculation, the culture was in the vegetative phase. **(C**,**D**) After 3 days, the culture was beginning aerial hyphae erection. **(E,F)** The culture was beginning sporulation 5 days after inoculation. **(G,H)** 8 days after inoculation, sporulation was visible on most of the surface. PE, phosphatidylethanolamine; OL, ornithine lipid; CL, cardiolipin; MLCL, monolyso-cardiolipin; DLCL, dilyso-cardiolipin; PIMs, phosphatidylinositol mannosides; UPL1, unknown phospholipid.

**FIGURE 6:**
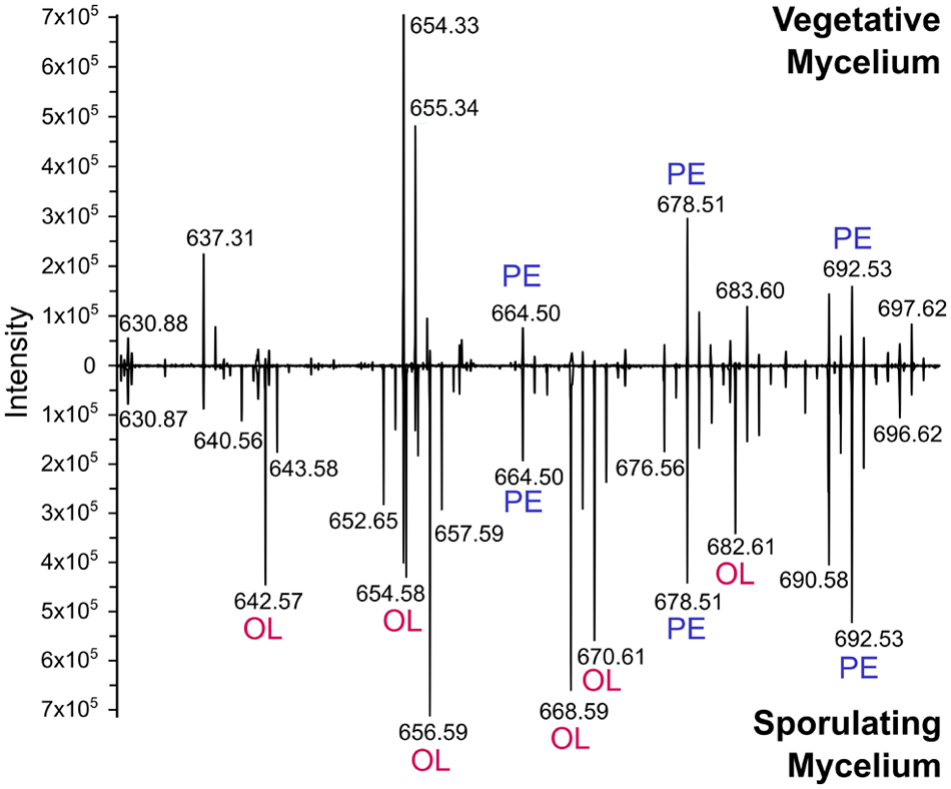
**Mass spectrometry analysis reveals accumulation of ornithine lipids in sporulating cultures.** Lipid extracts were obtained from *S. coelicolor* samples harvested at different developmental phases from cultures grown in SFM. The interval *m/z* 630–700 where PE and OL molecular ions can be detected is shown. After 2 days of incubation, the culture was in the vegetative phase, and only accumulating PE **(top)**. After 7 days of incubation, the culture was beginning sporulation and producing OL in addition to PE **(bottom)**. Identity of OL and PE precursor ions was assigned based on comparison of their MS/MS spectra (not shown) to reported fragmentation patterns of these lipids (Vences-Guzmán et al., 2011).

Several major differences were identified in the lipid composition from cultures harvested at different times after inoculation, even prior to the onset of morphological differentiation (**Figures 3A,B**). MS data suggested that OL (**9**) accumulation was correlated with specific developmental phases (**Figure 4**, inset). Therefore, lipid samples from *S. coelicolor* cultures grown in SFM solid medium and retrieved at different points during development were analyzed by TLC. In early vegetative mycelium PE (**3**), CL (**1**), MLCL (**4**), DLCL (**5**), and PIMs (**7**) were detected. At later stages of development we observed a sharp decrease in the content of PE (**3**) and a concurrent accumulation of OL (**9**). In the last samples taken from these media, PE (**3**) is absent and apparently replaced by OL (**9**) as the major zwitterionic lipid (**Figures 3C** and **5**). These results were corroborated with analysis of the mass spectra of lipid samples from vegetative and sporulating mycelium (**Figure 6**), where the molecular ions corresponding to OL (**9**) are detected only in the late cultures. Other differences observed between the lipid composition were a decrease in CL (**1**) and MLCL (**4**) at later time points, the accumulation of an unknown phospholipid that migrates with an R_f_ intermediate between DLCL (**5**) and PI, and an increase of different mannosylated derivatives of PI (**7**) (**Figures 3C** and **5**).

### OL and PE Accumulation are Dependent on P_i_ Availability in the Media

**FIGURE 7:**
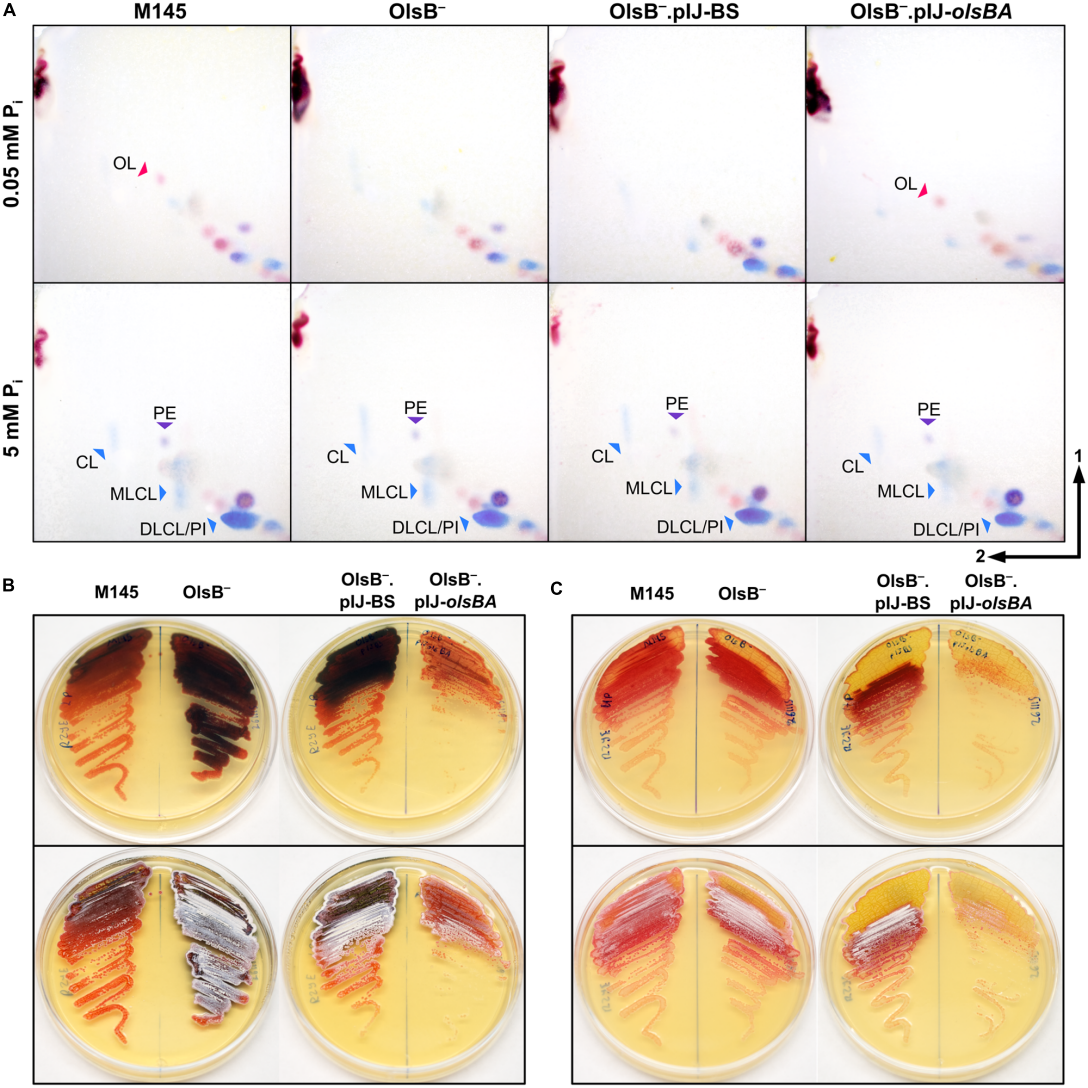
**Effects of phosphorus limitation in the lipid profile of *S. coelicolor* and phenotype of OlsB-deficient mutant.** **(A)** 2D-TLC analysis from the lipid extracts of *S. coelicolor* samples taken from cultures grown with different P_i_ concentrations in solid R2YE medium. Top panels show the lipid profiles of cultures grown under P_i_ limitation, and bottom panels show the lipid composition of cultures grown in high P_i_ concentrations. The strains compared are wild-type (M145), SCO0921-deficient mutant (OlsB^-^), the mutant with an empty vector (OlsB^-^.pIJ-BS), and the mutant with the *olsBA* operon in *trans* (OlsB^-^.pIJ-*olsBA*). TLCs were developed using system 1. Staining was performed with ninhydrin to reveal compounds containing primary amines (red spots, red arrowheads), and with phospray reagent to identify phosphorus-containing lipids (blue spots, blue arrowheads). Pictures of both stainings were overlaid, so amino-phospholipids are seen as dark violet spots (purple arrowheads). PE, phosphatidylethanolamine; OL, ornithine lipid; CL, cardiolipin; MLCL, monolyso-cardiolipin, DLCL, dilyso-cardiolipin, PI, phosphatidylinositol. **(B,C)** Plates of M145, OlsB^-^, OlsB^-^.pIJ-BS, and OlsB^-^.pIJ-*olsBA* strains after 60–72 h of incubation in R2YE solid medium with 0.05 mM P_i_ **(B)** or 5 mM P_i_ **(C)**. At the upper row, plates were photographed from below to show the production of actinorhodin (seen as dark blue shadows). At the bottom row, the same plates photographed from above showing the onset of aerial hyphae erection (seen as white over the orange-red vegetative mycelium).

In several bacterial species, OL (**9**) accumulation is induced by inorganic phosphate (P_i_) limitation (Hänel et al., 1985; Geiger et al., 1999). This led us to investigate the effect of P_i_ limitation on lipid composition in *S. coelicolor*. Cells were grown in R2YE solid medium with 0.05 and 5 mM P_i_ to test if OL (**9**) accumulation and PE (**3**) depletion were dependent on phosphorus content of the culture media. During vegetative growth at 0.05 P_i_ OL (**9**) accumulated, and PE (**3**) was not detectable (**Figures 3D** and **7A** top left panel). At 5 mM P_i_ OL (**9**) was not detectable but PE (**3**) was accumulated (**Figures 3D** and **7A** bottom left panel). The lipid profile under high phosphate conditions resembled the profile of vegetative mycelium samples grown in SMMS or SFM solid media (not shown). In addition to OL (**9**) accumulation, in the low P_i_ condition there was a higher content of unknown phosphorus-free amino-lipids than in the high P_i_ condition, as well as a relative lower amount of phospholipids (**Figure 7A**, left panels). The lipid profile of *S. coelicolor* grown in intermediate phosphate concentrations (0.39 and 0.5 mM P_i_) was very similar to the profile seen at 0.05 mM P_i_ (data not shown).

### The Putative *N*-acyltransferase Encoded by *SCO0921* is Required for Ornithine Lipid Biosynthesis

**FIGURE 8:**
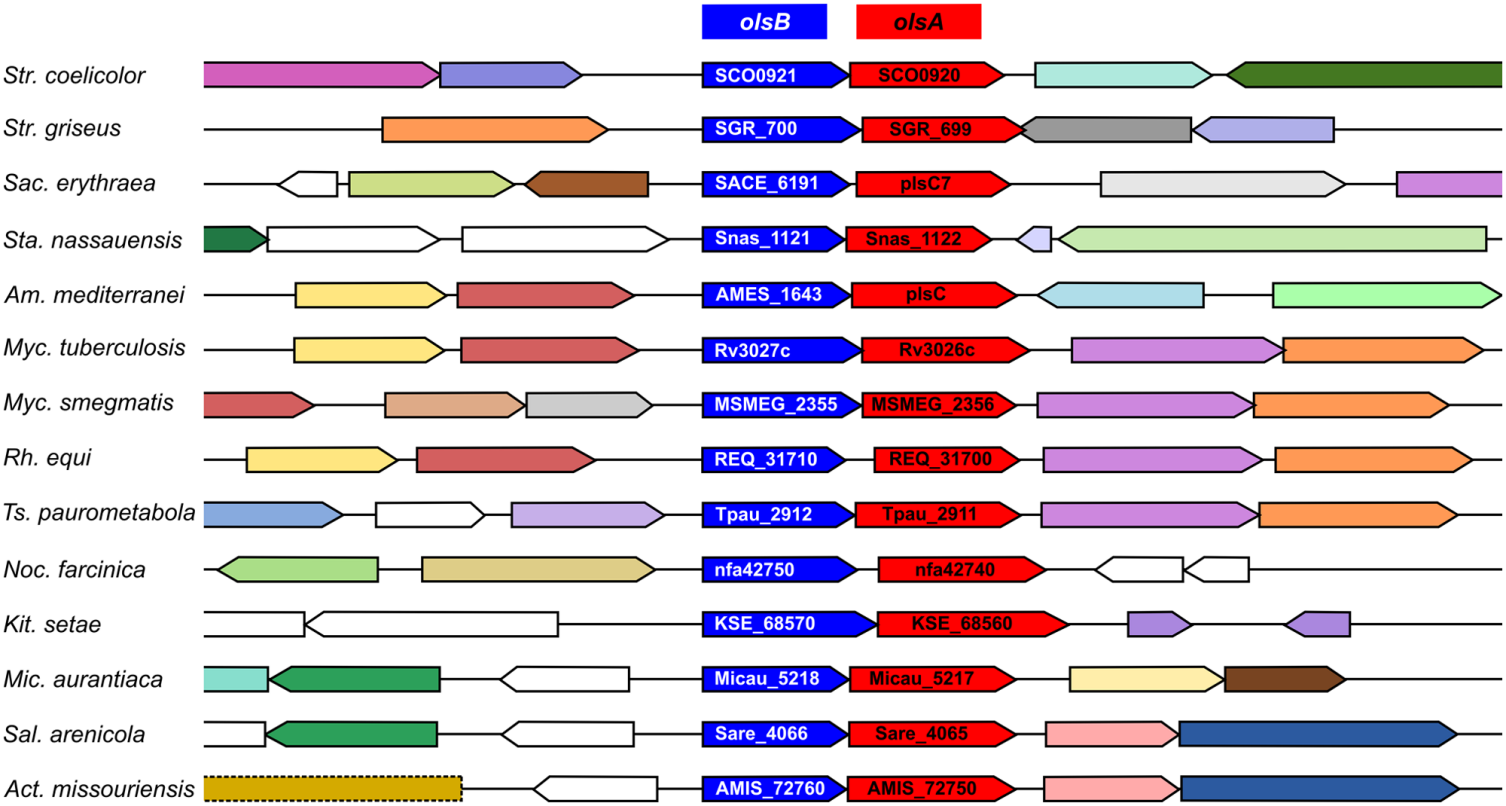
**The *olsB-olsA* putative operon is conserved in *Actinomycetes*.** Homologs of SCO0921, encoding putative *N*-acyltransferases (COG3176), are highlighted in blue. Homologs of SCO0920, coding for putative *O*-acyltransferases (COG0204), are indicated in red. The organisms compared are: *Streptomyces coelicolor* A3(2), *Streptomyces griseus* NBRC 13350, *Saccharopolyspora erythraea* NRRL 2338, *Stackebrandtia nassauensis* DSM 44728, *Amycolatopsis mediterranei* S699, *Mycobacterium tuberculosis* H37Rv, *Mycobacterium smegmatis* MC2 155, *Rhodococcus equi* 103S, *Tsukamurella paurometabola* DSM 20162, *Nocardia farcinica* IFM 10152, *Kitasatospora setae* KM-6054, *Micromonospora aurantiaca* ATCC 27029, *Salinispora arenicola* CNS-205, *Actinoplanes missouriensis* 431. The context analysis was performed with Gene Context Tool III (http://operons.ibt.unam.mx/gct3/). Genes are colored according to their COG.

Ornithine lipids (**9**) biosynthesis has been well characterized in α-proteobacteria (Weissenmayer et al., 2002; Gao et al., 2004), β-proteobacteria (González-Silva et al., 2011), and γ-proteobacteria (Vences-Guzmán et al., 2014). A BLAST search in the genome of *S. coelicolor*, using OlsB from *Sinorhizobium meliloti* as a query, resulted in a hypothetical protein with an *N*-acyltransferase domain (SCO0921: 35% identity, 49% similarity, 82% coverage). There is a gene encoding an *O*-acyltransferase (*SCO0920*) apparently forming part of the same operon, indicating that SCO0920 could be homologous to OlsA (33% identity, 44% similarity, 81% coverage), the second enzyme in the OL (**9**) biosynthetic pathway. Both genes are conserved with the same organization across the actinomycetes (**Figure 8**). Furthermore, these genes were overexpressed in *S. coelicolor* under P_i_ limitation conditions (Martín et al., 2012).

A mutant deficient in *SCO0921* was constructed to verify the role of these putative enzymes in the synthesis of OL (**9**). Inactivation of *SCO0921* was accomplished by replacing the wild-type copy with an interrupted copy bearing an insertion of transposon Tn*5062*. As expected, the mutant strain was incapable of OL (**9**) accumulation (**Figure 7A**), and expression in *trans* of the *SCO0921-SCO0920* operon restores wild-type OL (**9**) production (**Figure 7A**, top right panel). *SCO0921* is thus renamed *olsB*, and *SCO0920*, *olsA*. The rest of the lipid profile beyond OL (**9**) is similar when comparing M145 (wild-type) and mutant strains grown in R2YE solid medium.

### OlsB-Deficient Mutant Presents Precocious Development and Actinorhodin Production

OlsB^-^ mutant began production of the blue pigment γ-actinorhodin earlier than the wild-type strain in R2YE solid medium under P_i_ limitation (**Figure 7B**, top panel), and maintained a higher production of this compound throughout development. There was no apparent difference between the strains in actinorhodin production at 5 mM P_i_ (**Figure 7C**, top panel). The OlsB-deficient mutant also displayed an accelerated onset of morphological differentiation, beginning aerial hyphae erection and sporulation earlier than the WT strain (**Figures 7B,C**, bottom panels). This effect was more evident in the low P_i_ conditions, but there was also a visible difference in high P_i_ conditions. The OlsB^-^ mutant complemented with the *olsBA* operon presented delayed pigment production and morphological development when compared with the mutant carrying an empty vector (**Figures 7B,C**). Precocious development of the OlsB-deficient strain was also apparent in SMMS and SFM solid media (data not shown).

## Discussion

Lipid composition of bacterial membranes has long been used as part of the description and classification of new species (Shaw, 1974; Lechevalier et al., 1977; Tindall et al., 2010; Zhang et al., 2011). This was based on the assumption that related organisms would share core metabolic activities and, therefore, would be able to synthesize similar membrane lipids. While this premise is basically correct, the second proposition being assumed is that the lipidic composition of bacteria would be mostly stable throughout different growth conditions. This latter assumption is clearly inaccurate and does not take into account the enormous complexity of the cellular activities of bacterial membranes, nor the active role they play in the response to environmental and nutritional stress (Sohlenkamp and Geiger, 2015). The adaptation of the membrane lipid composition to diverse environmental challenges has been thoroughly reported in a wide range of bacterial genera. While some model organisms like *E. coli* do not show major qualitative differences in their lipid profiles (Shaw, 1974), some other bacterial species can drastically alter their membrane lipid components as a result of nutritional stress (Geiger et al., 1999; Sohlenkamp and Geiger, 2015).

Here we describe a drastic difference in lipid composition between samples extracted from *S. coelicolor* cells grown in liquid or solid media with almost identical nutritional conditions. In earlier studies *S. coelicolor* was grown in liquid medium, but it must be noted that dispersed growth in liquid medium is a very artificial growth condition for streptomycetes. These bacteria normally grow as multicellular mycelium on soil, either on surfaces or the aqueous/aerobic interphase. It is plausible that the constant stress of the forced fragmentation of mycelium pellets that is needed to achieve somewhat dispersed growth in liquid medium, imposes special constraints in membrane properties that are best fulfilled by the lipid species seen accumulated in this condition.

When growing on a selection of solid media, the lipid composition of *S. coelicolor* showed surprising differences. In some cases these differences might be explained by nutrient availability, pH, and osmolarity, which vary across the media tested (**Supplementary Table S1**). However, as was the case for YEME liquid vs. solid media, it is surprising that even in media with similar compositions – like ISP2 and YEME solid media – vegetative cultures had very contrasting lipid profiles (**Figures 2** and **3A,B**). An unexpected correlation was found between undecylprodigiosin production in the cultures grown in solid media and DLCL accumulation in the membranes. An adaptation of the membrane composition as a response to the accumulation of this hydrophobic secondary metabolite could explain some similarities in the lipid profiles from cultures grown in media with different compositions such as solid YEME and R2YE. Additionally, while there were several differences in the lipid profiles from early and late vegetative cultures, there was much less variation between these two time points than across the different media tested (**Figures 3A,B**).

We also investigated whether the membrane composition changed during morphological differentiation. One of the most relevant changes was the disappearance of PE, and its apparent substitution with OL in samples taken at the later stages of development. It had been previously reported that OL was accumulated in *Streptomyces viridochromogenes* after a long incubation time, and that this effect was associated with P_i_ concentration in culture medium (Shim and Kim, 1993), however, the complete replacement of PE observed in our results (**Figures 3C** and **5**) is much more drastic than what had been reported in other actinomycetes. We show that OL accumulation is induced by phosphate limitation. While phosphate depletion of the media after prolonged growth is a likely explanation for the accumulation of OL in sporulating cultures of *S. coelicolor*, it is possible that OL is involved in some other cellular process during differentiation.

We identified the genes responsible for OL synthesis in *S. coelicolor*, *SCO0921* (*olsB*) and *SCO0920* (*olsA*). These genes apparently constitute an operon and are induced under P_i_ limitation (Martín et al., 2012). Homologs of these genes are widely distributed across many actinomycetes (**Figure 8**), including *Mycobacterium tuberculosis* (Rv3027 and Rv3026). It is known that *M. tuberculosis* accumulates OLs (Lanéelle et al., 1990), but the biosynthetic genes for OL formation had not been identified in any species of actinobacteria. A *S. coelicolor* mutant deficient in OlsB, besides being unable to synthesize OL, had an altered secondary metabolism production and development. It is assumed that under phosphate starvation conditions, some bacteria replace their phospholipids with phosphorus-free lipids, in order to direct the limited phosphate to core cellular functions such as nucleic acid synthesis (Geiger et al., 1999). In *Streptomyces*, there is also a known link between P_i_ availability and secondary metabolism, with several species overproducing antibiotics as a result of P_i_ limitation, and repressing their synthesis when there is P_i_ abundance (Doull and Vining, 1990; Sola-Landa et al., 2003; Santos-Beneit et al., 2009). We propose that precocious actinorhodin production in the OlsB-deficient mutant is a response to alterations in phosphate metabolism of this strain when grown in P_i_ limitation conditions. The difference in onset of development seen in this mutant, however, proves harder to explain just as a result of phosphate starvation. While evidence exist regarding nutrient depletion as a cue for triggering differentiation in *Streptomyces*, most that has been reported involves carbon and nitrogen sources (McCormick and Flärdh, 2012). A difference in the membrane properties of the OlsB-deficient mutant could not be ruled out as an explanation for the accelerated morphological development.

Our results clearly show the plasticity of membrane lipid composition in *S. coelicolor* and form a starting point for investigations regarding the role of membrane composition dynamics in development and stress response in streptomycetes.

## Conflict of Interest Statement

The authors declare that the research was conducted in the absence of any commercial or financial relationships that could be construed as a potential conflict of interest.

The reviewer Xiaoqiang Jia and handling Editor declared their shared affiliation, and the handling Editor states that the process nevertheless met the standards of a fair and objective review.

## Footnotes

1 http://gnps.ucsd.edu/ProteoSAFe/static/gnps-splash.jsp

2 www.cytoscape.org

## Abbreviations

CL: cardiolipin
DLCL: dilyso-cardiolipin
MLCL: monolyso-cardiolipin
OL: ornithine lipid
PA: phosphatidic acid
PE: phosphatidylethanolamine
PG: phosphatidylglycerol
PI: phosphatidylinositol
PIMs: phosphatidylinositol mannosides
TLC: thin layer chromatography
MS: mass spectrometry
