# Supplementary material for: Plasticity of Streptomyces coelicolor Membrane Composition Under Different Growth Conditions and During Development
Source: Front Microbiol. 2015 Dec 22;6:1465. doi: 10.3389/fmicb.2015.01465 (PMC4686642; doi:10.3389/fmicb.2015.01465)
Supplement: Supplementary file 1 [file Table_1.DOCX]

**Table S1. Composition of culture media used in this work.**

| **Media** | **Complex nutrients** | **Main carbon source** | **P_i_** | **Sucrose** | **Buffer** | **Other components** |
| --- | --- | --- | --- | --- | --- | --- |
| **YEME**  **liquid** | Yeast extract, Malt extract, Peptone. | Dextrose |  | 34% |  |  |
| **YEME**  **solid** | Yeast extract, Malt extract, Peptone, Agar. | Dextrose |  | 30% |  |  |
| **ISP2**  **solid** | Yeast extract, Malt extract, Agar. | Dextrose |  |  |  |  |
| **SFM**  **solid** | Soy Flour, Agar. | Mannitol |  |  |  | Tap water |
| **R2YE**  **solid** | Yeast Extract, Casaminoacids, Agar. | Dextrose | 0.39 mM | 10% | Y | MgCl_2_, CaCl_2_, K_2_SO_4_, Proline, Trace elements,  NaOH |
| **SMMS**  **solid** | Casaminoacids, Agar. | Dextrose | 1 mM |  | Y | MgSO_4_, Trace elements |
