# Supplementary material for: Plasticity of Streptomyces coelicolor Membrane Composition Under Different Growth Conditions and During Development
Source: Front Microbiol. 2015 Dec 22;6:1465. doi: 10.3389/fmicb.2015.01465 (PMC4686642; doi:10.3389/fmicb.2015.01465)

Supplementary Figure 1

Figure S1. Separation of lipid standards by 2D-TLC. Phospholipid standards were developed separately using the solvent systems for first dimension [chloroform/methanol/water (16:4:1, v/v)] and second dimension [chloroform/ethanol/water/triethylamine (30:35:7:35 v/v)] of System 2, or mixed and developed in a 2D-TLC. TLCs were stained with Phospray reagent (Supelco) to reveal phospholipids. Standards used were: PI, phosphatidylinositol (Sigma), 8 µg; PG, phosphatidylglycerol (Sigma), 8 µg; PA, phosphatidic acid (Sigma), 4 µg; PE, phosphatidylethanolamine (Sigma), 6 µg; DLCL, dilyso-cardiolipin (Avanti Polar Lipids), 10 µg; MLCL, monolyso-cardiolipin (Avanti Polar Lipids), 10 µg; CL, cardiolipin (Sigma), 16 µg.

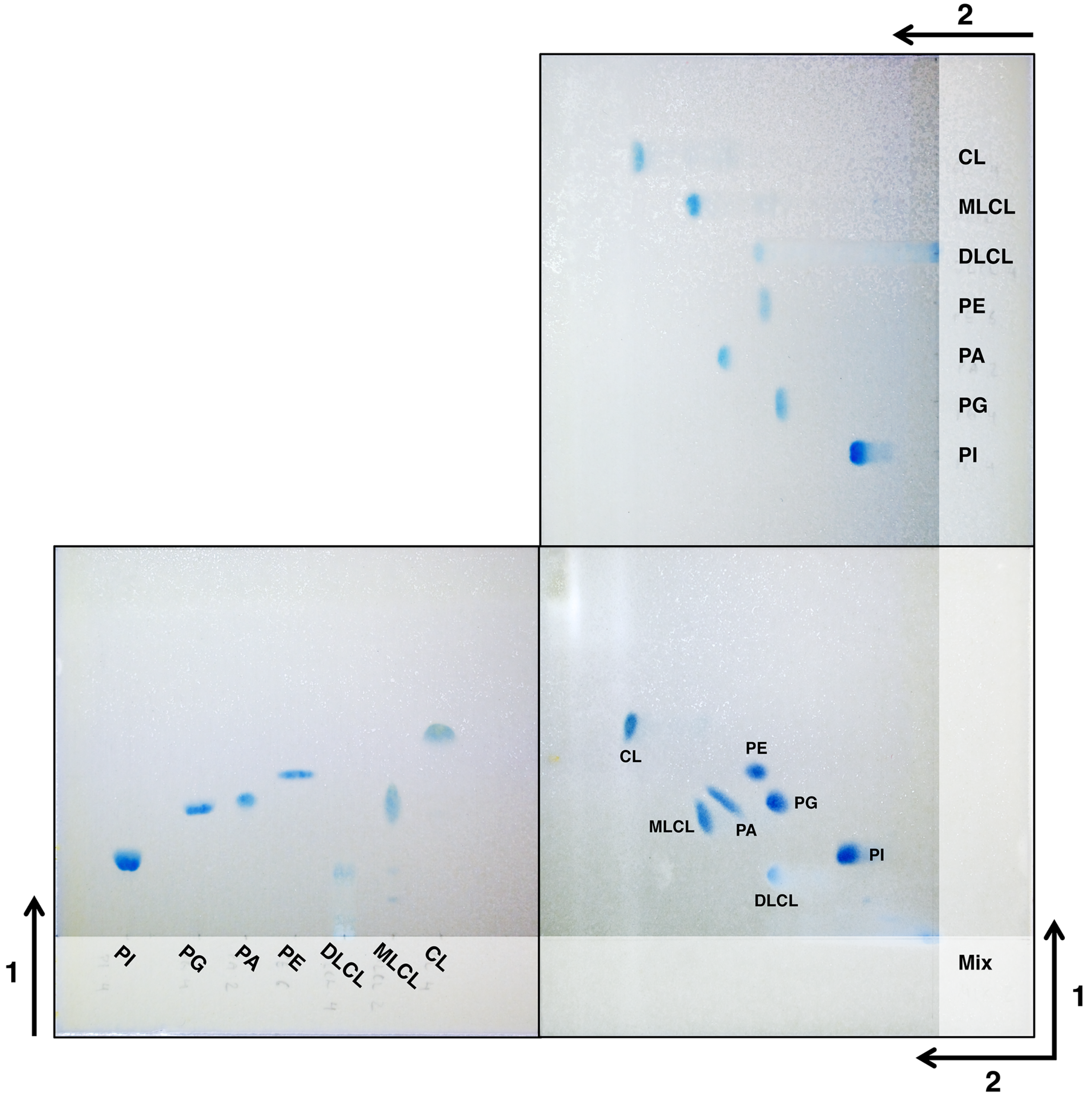

Supplement: Supplementary file 2 [file Image_1.PDF]
