# Supplementary material for: Plasticity of Streptomyces coelicolor Membrane Composition Under Different Growth Conditions and During Development
Source: Front Microbiol. 2015 Dec 22;6:1465. doi: 10.3389/fmicb.2015.01465 (PMC4686642; doi:10.3389/fmicb.2015.01465)
Supplement: Supplementary file 3 [file Image_2.PDF]

## Supplementary Figure 2

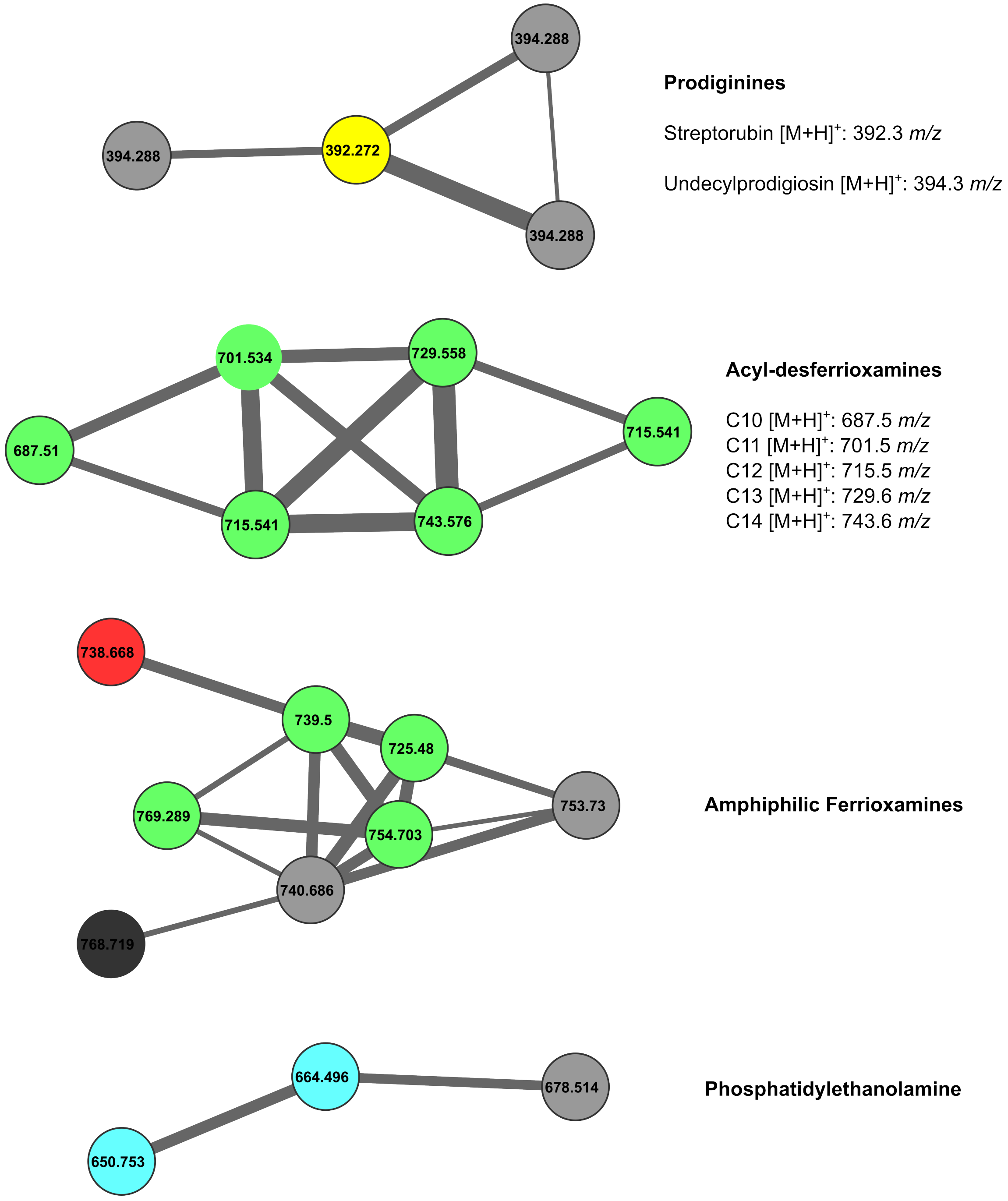


**Figure S2. Selected clusters from the molecular network of *S. coelicolor* lipid extracts.** The annotated clusters from **Figure 4** are presented separately and with additional information. Nodes represent different precursor ions in the samples, and edges between the nodes represent the similarity between fragmentation spectra of the corresponding precursor ions. Node colors denote different culture media. Annotation was performed based on the cosine scores with matches from compounds in the GNPS library and comparison of their MS/MS profiles, or by comparison with MS/MS fragmentation reported in the literature.
